# Advanced risk and hazard analysis in the egg sorting-packing units industry from supplier selection to delivery in chain stores under global food safety initiative integrated food safety programs

**DOI:** 10.1016/j.heliyon.2025.e42587

**Published:** 2025-02-11

**Authors:** Ioana Cristina Crivei, Romina Alina Marc, Roxana Nicoleta Rațu, Crina Carmen Mureșan, Alina Narcisa Postolache, Florina Stoica, Ionuț Dumitru Veleșcu, Aida Albu

**Affiliations:** a Department of Food Technology, Faculty of Agriculture, “Ion Ionescu de la Brad” University of Life Sciences, 3 Mihail Sadoveanu Alley, 700490, Iasi, Romania; b Food Engineering Department, University of Agricultural Science and Veterinary Medicine Cluj-Napoca, Faculty of Food Science and Technology, 3-5 Calea Mănăştur Street, 400372, Cluj-Napoca, Romania; c Department of Pedotechnics, Faculty of Agriculture, “Ion Ionescu de la Brad” University of Life Sciences, 3 Mihail Sadoveanu Alley, 700489, Iasi, Romania; d GFSI (IFS, FSSC22000) International Lead Auditor & Consultant, Romania; e Control, expertise and services Department, Faculty of Food and Animal Sciences, “Ion Ionescu de la Brad” University of Life Sciences, 3 Mihail Sadoveanu Alley, 700490, Iasi, Romania

**Keywords:** GHP, HACCP, ISO 22000, IFS food, GFSI, Food safety management

## Abstract

This study outlines a comprehensive examination of risks and hazards in three egg sorting and packing units, covering the entire process from supplier selection and evaluation to egg delivery in chain stores. The analysis is conducted within the framework of Codex Alimentarius and GFSI-integrated food safety programs. Salmonella is the greatest significant threat. To enhance the Hazard Analysis of Critical Control Points (HACCP), preparatory programs were incorporated into the quality management system (QMS) by monitoring and assessing the biological, chemical, and physical threats according to Code Alimentarius and further integrated into GFSI food safety programs, including food authenticity and food defense. The procedure offered sophisticated preventive tools, hand-on at any time, for eliminating, reducing, or mitigating the risks encountered in egg packing and sorting facilities.

## Graphical abstract

**Figure:**
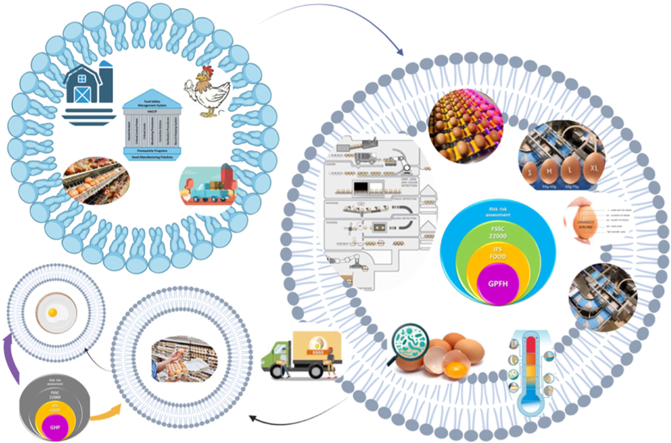

## 1 Introduction

There is an increasing demand among consumers for eggs from farms that prioritize animal welfare. Consumers perceive eggs produced in non-cage environments as food that not only meets ethical standards but also provides improved acceptance, nutritional content, and taste. The table egg is the most affordable type of animal protein, rich in nutrients, and has only 75 calories per egg. Due to their optimal amino acid composition and efficient digestion, they serve as a highly commendable protein source for human consumption [1].

Laying hens can consume contaminants such as dioxins, heavy metals, dioxins, dl-PCBs, cleaning and sanitizing chemicals, or veterinary pharmaceutical treatments from the environment, water, soil, and feed [2]. These contaminants can then be transferred into the eggs. Due to their elevated fat content, eggs can harbor a substantial amount of persistent organic pollutants (POPs), such as dioxins and polychlorinated biphenyls (PCBs), which pose potential health risks to individuals. *Salmonella* spp. is the most prevalent pathogen associated with eggs and egg products. Additional pathogens that become significant when the process of egg production is transformed into liquid egg products encompass *Bacillus cereus* and *Listeria monocytogenes* [3].

Conventional methods are ineffective in managing initial process risks to guarantee the hygienic integrity of the end goods [4]. To maintain food safety, it is imperative to adhere to Good Manufacturing Practices (GMP), Good Hygiene Practices (GHP), and the principles of Hazard Analysis of Critical Control Points (HACCP) [5]. The objectives of HACCP systems are to identify, evaluate, and control risks. ISO 22000 has not been adopted as a standard reference for food manufacturers by the Global Food Safety Initiative (GFSI) because of its lack of relevant PRP (prerequisite program) information. ISO22000:2018 provides improvements that largely concentrate on the identification of a PRP (pre-requisite program) and the CCP (critical control point) for key risks, employing risk-based thinking and risk reduction as guiding principles [6,7].

Recent studies have highlighted significant risks associated with persistent organic pollutants (POPs) such as dioxins and PCBs, which are frequently detected in eggs due to environmental contamination. Advanced analytical methods like LC-MS/MS have enhanced the detection of these pollutants at trace levels, emphasizing the need for stringent monitoring programs [8]. Furthermore, pathogen management, particularly addressing Salmonella spp., remains a critical focus, as evidenced by outbreaks linked to inadequate control measures [9]. Current global food safety standards, such as Codex Alimentarius (2023) and FSSC 22000 v6, have introduced updated frameworks that integrate risk-based thinking, offering more robust preventive strategies [10]. These developments underscore the importance of continuous improvement in egg safety protocols, aligning with consumer demand for ethically produced, high-quality eggs [7].

Emerging contaminants, such as perfluoroalkyl substances (PFAS), are increasingly being recognized as significant risks to egg safety. Studies have shown that even trace levels of these substances can persist in eggs due to their bioaccumulative nature, posing potential health risks [11]. Incorporating advanced detection methods, like LC-MS/MS, has allowed researchers to assess contamination at sub-ng/kg levels, underscoring the importance of routine surveillance to safeguard food quality [12].

Egg safety directly impacts public health and economic stability. Outbreaks linked to pathogens such as Salmonella not only endanger consumer health but also result in significant economic losses due to product recalls and trade restrictions [13]. Moreover, growing consumer awareness and stringent regulatory frameworks have driven producers to adopt advanced food safety management systems that encompass food defense and fraud prevention measures [14].

Innovative approaches in risk management, such as the integration of machine learning algorithms and predictive modeling, are becoming pivotal in the egg safety domain. These tools enhance the ability to anticipate contamination events and implement proactive measures, reducing reliance on post-contamination responses [15]. Furthermore, blockchain-based traceability systems are gaining traction for ensuring transparency and accountability across the supply chain [7].

The alignment of egg safety protocols with sustainability goals has garnered attention in recent years. Sustainable practices, including the reduction of waste through efficient egg grading systems and the minimization of energy consumption during storage and transport, are becoming integral to global food safety standards [9]. Such measures not only protect public health but also contribute to environmental conservation.

The objective of this study was to comprehensively examine the entire process involved in the selection and evaluation of suppliers, as well as the delivery of eggs to chain stores. This included conducting a detailed analysis of risks and hazards in three specific facilities related to egg sorting and packaging. The study focused on establishments that had implemented various food safety management systems. The objective of this research is to facilitate the exchange of technological information to benefit both egg safety scientists and the economic environment.

## 2 Materials and methods

### 2.1 Materials

The research was carried out at three different egg sorting and packing facilities located in different counties from Romania, with the selection criteria based on geographical dispersion, operational size, and production capacity. Sampling included the collection and analysis of 300 eggs (100 eggs/unit). Sampling was conducted in accordance with the principles of stratified random sampling, ensuring that both environmental factors, including temperature and humidity, and egg handling procedures at all three sorting and packing locations were thoroughly documented.

Eggs were chosen based on size (50–60 g), weight uniformity, and absence of visible defects. Sampling criteria also included traceability to specific production batches and compliance with EU Regulation 589/2008.

Biological, chemical, and physical analyses were performed on the collected samples. Contaminant levels, including dioxins, PCBs, and heavy metals, were quantified using LC-MS/MS and GFAAS techniques. Microbiological analyses for the detection of *Salmonella* spp. were performed using ISO 6579:2017 methods. Antibiotic residues were assessed with the Charm II system, in accordance with EU Regulation 37/2010.

The data were analyzed using one-way ANOVA to compare differences in contaminant levels and pathogen prevalence across egg sorting and packaging facilities. Post hoc Tukey's tests identified significant group differences at p < 0.05. All analyses were conducted using SPSS v27. Quality assurance included triplicate sample analyses and daily instrument calibration against certified standards.

### 2.2 Methods

The Prerequisite Programs (PRPs) were performed according to Cusato's methods and in compliance with the provisions of the Codex Alimentarius of 2023 [[10], [16], [17]].

Residues of antibiotics were detected using the Charm II System, for Beta Lactams (in compliance with EU Reg. 1644/2022, DC 657/2002) and for Macrolides Charm II System, (in compliance with EU Reg. 1644/2022, DC 657/2002/EC Reg. 37/2010/EC). Antimicrobial residues (b-lactams, macrolides, and tetracyclines) were qualitatively detected using the Charm II [14,18]. The relative humidity, yolk and white pH values, and temperature were determined according to the guidelines set by the EFSA BIOHAZ Panel [3]. Heavy metal residues have been determined using Graphite furnace atomic absorption spectrometry (GFAAS) technique [19]. Dioxin residues have been detected using the method presented by Ten Dam G. et al. [20]. Perfluoroalkyl and polyfluoroalkyl substances were quantitatively determined using LC-MS/MS technique [8]. As f for melamine determination, LC-MS/MS technique [11], was used. Fipronil residues have been assessed via LC-MS/MS analysis [12].

Descriptive statistics were used to calculate means and standard deviations for relative humidity, temperature, and pH. To assess the variability in microbial contamination, dioxin residues, and relative humidity across facilities, ANOVA test was employed. Regression analysis explored the correlation between environmental factors (e.g., temperature, humidity) and key quality indicators (air cell size, microbial load).

## 3 Results

The egg sorting and packaging procedure comprises several steps to guarantee the quality, integrity, and precise classification of the eggs. After delivery and initial storage, the eggs undergo additional quality assessment. They are subsequently evaluated based on their size and weight. The egg packing procedure entails meticulously organizing them in cartons or trays to ensure their safety and facilitate transportation. They are thereafter preserved in a regulated environment, where temperature and humidity are controlled, until delivery. Thus, it is ensured that the eggs remain fresh and safe for human consumption.

The criteria for accepting eggs in the sorting and packaging units encompass several factors, including the overall health of the poultry flock (such as the presence of pathogens), the level of contaminants on or within the eggs, the usage of agricultural and veterinary chemicals, the eggs freshness, the handling protocols, and any treatments used to kill microorganisms.

Table 1 outlines the risk assessment framework and food safety management systems employed at the three examined egg sorting and packaging facilities (units A, B, and C). They function in accordance with the requirements of different Global Food Safety Initiative (GFSI) schemes and standards, including GPFH (GHPs, HACCP), IFS Food v8, and FSSC 22000 v6. The table illustrates the implementation of risk assessment procedures at each location, adapted to the specific GFSI scheme employed. Consequently, each facility mitigates the risks of contaminants to guarantee the safety of table eggs; cleaning and disinfection are critical prerequisite programs at all locations, alongside detailed descriptions of products and processes within their hazard control plans. Hazards are categorized as physical, chemical, or biological, originating from either unintentional or intentional sources. Additional considerations include control measures and limits, validation and verification, records and traceability, fraud and threat Assessments, along with incident management and recall of non-conforming products.

**Table 1:** Elements of the risk assessments applied in the studied egg sorting and packaging units A, B, C, according with GPFH [GHPs, HACCP], FSSC 22000, and IFS Food scheme requirements.

Item	Requirements		
	2 GFSI schemes		UnitC: GPFH [GHPs HACCP, v. 2023]
	Unit A: IFS Food v8, April 2023	Unit B: FSSC 22000 v6, April 2023	
Contaminants	- food safety and food quality in PRPs;	- food safety and food quality in ISO 22002-X;	- food safety and suitability in GHPs;
Cleaning and disinfection	- PRPs	- ISO 22002-X;	- GHPs and higher focus;
Product description	- HACCP;	- hazard control plan;	- GHPs;
Process description	- HACCP;	- hazard control plan;	- GHPs;
Operational control	- HACCP;	- hazard control plan;	- GHPs;
Operational monitoring	- PRPs;	- ISO 22002-X;	- GHPs;
Corrective actions in case of process failure	- PRPs;	- ISO 22002-X;	- GHPs;
Validation	- PRPs and cleaning;	- ISO 22002-X and cleaning;	- GHPs and cleaning;
Verification	- PRPs;	- ISO 22002-X;	- GHPs;
Records	- PRPs;	- ISO 22002-X;	- GHPs;
Hazards	- physical [metal, plastic, hard plastic, etc.]; - chemical [inclusive allergens, radioactivity, contaminants as melamine, fipronil, heavy metals, eggs fraud, etc.]; - biological [*Enterobacteriaceae*, *Salmonella* spp];	- physical [metal, plastic, hard plastic, etc.]; - chemical [inclusive allergens, radioactivity, contaminants as melamine, fipronil, heavy metals, eggs fraud, etc.]; - biological [*Enterobacteriaceae*, *Salmonella* spp];	- physical [metal, plastic, hard plastic, etc.]; - chemical [inclusive allergens, radioactivity, contaminants as melamine, fipronil, heavy metals, eggs fraud, etc.] biological [*Enterobacteriaceae*, *Salmonella* spp].
Hazard sources	- unintentional [farms, transport]; - intentional [economic motivation gain related with egg fraud – mislabeling];	- unintentional [farms, transport]; - intentional [economic motivation gain related with egg fraud – mislabeling];	- unintentional [farms, transport];
Occurrence in absence of control	- hazard analysis as HACCP Codex Alimentarius 2023;	- hazard analysis as ISO 22000 requirements;	- hazard analysis [3x3 matrix];
Severity in absence of control	- hazard analysis as HACCP Codex Alimentarius 2023;	- hazard analysis as ISO 22000 requirements;	- hazard analysis [3x3 matrix];
Significant hazard	- hazard analysis as HACCP Codex Alimentarius 2023 [with scientific or industry practice justification];	- hazard analysis as ISO 22000 requirements [with scientific or industry practice justification];	- simple qualitative hazard analysis with scientific or industry practice justification
Control measure	- point of attention/control point; - critical control point;	- operational prerequisites program; - critical control point;	- critical control point;
Control limit	- observable and/or measurable parameters;	- observable parameters for OPRP and measurable parameters for OPRP or CCP;	- observable and measurable parameters;
Limit control definition	- critical limit;	- action criteria; - critical limit;	- critical limit;
Monitoring	- critical limit;	- action criteria; - critical limit;	- critical control point;
Correction	- direct action [immediately];	- in time/promptly action for critical control point;	- not mentioned;
Corrective action	- root cause analysis and prevention of recurrence;	- root cause analysis and prevention of recurrence;	- critical control point;
Validation	- critical control point;	- operational prerequisites program; - critical control point;	- critical control point;
Verification	- calibration; - raw material [eggs], - packaging materials [carboard working forms and PET casseroles], in process product, finish product testing; - environmental testing; - monitoring; - corrective action;	- calibration; - raw material [eggs], - packaging materials [carboard working forms and PET casseroles], in process product, finish product testing; - environmental testing; - monitoring; - corrective action;	- calibration; - raw material [eggs], - packaging materials [carboard working forms and PET casseroles], in process product, finish product testing; - environmental testing; - monitoring; - corrective action;
Test reports	- on annual base; - at changes;	- on annual base; - at changes;	- appropriate period - at changes;
Records keeping	- one year + egg shelf life [28 days from lying period]	- one year + egg shelf life [28 days from lying period];	- appropriate period;
Recall	- in the management system part	- in the management system part;	- GHPs;
Input raw material and auxiliary risk assessment	- hazard analysis [3x3 matrix] at reception step	- hazard analysis [3x3 matrix] at reception step;	- hazard analysis [3x3 matrix] at reception step;
Fraud assessment	- fraud occurrence, detection based on criteria established by the company [f.e.: history, economic gain, access to supply chain, the possibility to be frauded – nature of the product, credibility of the suppliers, etc.]	- fraud occurrence, detection based on criteria established by the company [f.e.: history, economic gain, access to supply chain, the possibility to be frauded – nature of the product, credibility of the suppliers, etc.]	- not mentioned;
Threat assessment/Food defense	- emphasize the evaluation of specific areas and the consequences of success, such as suspending production, causing harm to the company/product [egg], and consumer health;	- emphasize the evaluation of specific areas and the consequences of success, such as suspending production, causing harm to the company/product [egg], and consumer health;	- not mentioned;
Supplier control	- supplier selection and evaluation or HACCP;	- supplier selection and evaluation or HACCP;	- GHPs and HACCP;
Incoming inspection	- incoming inspection;	- Incoming inspection	- GHPs and HACCP;
Quality control	- PRPs; - product safety and quality operational control plan;	- as ISO 9001;	- GHPs;
Incident management	- centered on intentional occurrence, sabotage, and cyberattack, connected to the recall process;	- covered by the requirements for emergency preparedness and response;	- not mentioned;

### 3.1 Assessment and implementation of the PRPs

An evaluation was conducted to analyze the application of PRPs in relation to buildings, facilities, equipment, utensils, food handlers, production, food transportation, and documentation. Following the evaluation and identification of deviations, operational protocols were implemented. Training in the deployment of food safety systems is the crucial stage. Observations were made on the implementation of theoretical and practical training to improve practices and behaviors related to Good Manufacturing Practices (GMP) and Good Hygiene Practices (GHP) [15].

### 3.2 Implementation of HACCP plan

The implementation of the HACCP plan respects the 12 essential steps.

Preliminary steps to enable hazard analysis (Step 1–6) include.

#### 3.2.1 Food safety teams

The teams responsible for ensuring the quality and safety of the eggs in the 3 units are multidisciplinary, thoroughly trained and are made up of: HACCP team leader, technological engineer, test laboratory head, hygiene manager, mechanical engineer, supply manager, distribution manager, HACCP team secretary.

#### 3.2.2 Specifications and intended purpose of the product

Prior to providing a comprehensive description of the eggs, the food safety team identified their exact composition as stated in the technical sheet. Table 2 provides a concise overview of the attributes of eggs and their suggested use for all population segments, except individuals who are sensitive due to egg allergies (qualitative characteristics, physical and chemical characteristics, microbial conditions, residues, details regarding marking and packaging, storage, transport, documentation, and shelf life)

**Table 2:** Eggs product description.

Specification	Description	Mentions
Product name	Eggs – category A	
Technical quality conditions	The eggs come from hen farms that are sanitary and veterinary-authorized for consumption. Laying hens are raised in batteries or on the ground in compliance with the legal requirements regarding their welfare.	
Qualitative characteristics	Shell and cuticle: clean, intact, normal; Air cell: the height does not exceed 6 mm, immovable; however, for eggs marketed with the mention "extra", it must not exceed 4 mm; Yolk: visible in the beam of light only as a shadow, without a precise outline; when the egg is turned, the yolk is slightly mobile and returns to the central position; Albumen: clear, translucent; Foreign bodies: no foreign bodies; Foreign odor: no foreign odor.	Tolerances for category A quality defects: At the packing center, just before shipping, 5 % of the eggs have quality defects; In the other stages of marketing, 7 % of the eggs have quality defects; For eggs with the mention "extra", no tolerance for the height of the air chamber is allowed during the inspection carried out during packaging; The percentages are doubled when the controlled lot contains less than 180 eggs.
Classification of eggs according to weight	XL - very large - weight greater than or equal to 73 g; L - large - weight less than 73 g and greater than or equal to 63 g, M − medium - weight less than 63 g and greater than or equal to 53 g; S - small - weight less than 53 g	Tolerances for egg weight A batch can contain no more than 10 % of eggs from the weight categories close to the one marked on the package, but no more than 5 % from the weight category immediately below. When eggs of different sizes are packed in the same package, the minimum net weight of these eggs is indicated in grams, and the mention "eggs of different sizes" is applied on the outside of the package. Category A eggs are neither washed nor cleaned, neither before nor after classification. Eggs should not be washed or cleaned, as this can cause damage to the shell, which due to its antimicrobial characteristics represents an effective barrier against bacterial contamination.
Physical - chemical characteristics	The protein content of the albumen: 11–12 % pH albumen: 7.8–9.3 The protein content of the yolk: 16–17 % pH yolk: 5.6–7	
Microbiological conditions	Salmonella (Spp/25 g): absent	According Reg. 1441/2007 [30]
Maximum contaminant limits	Sum of dioxin – max 2.5 pg/g fat Sum of dioxins and dioxin-like PCBs – max 5.0 pg/g fat	According Reg. 915/2023 [31]
Residues of medicine	≤200 - Chlortetracyclin, Oxytetracycline, Tetracycline, Tylosin; ≤150 - Erythromycin; ≤400 - Neomycin; ≤1000 - Tiamulin	According to DC 657/2002/EC [32]; Reg. 37/2010 [33]
Residues of pesticides	absent	According to Reg. 396/2005 [34]; Reg. 710/2023 [35]; Reg. 1049/2023 [36]; Reg. 1042/2023 [37]
Radioactive contamination	absent	According to Reg. 52/2016 [38]
Melamine	max 2.5 mg/kg	According to Reg. 915/2023 [31]
Rules for checking quality	Checking the quality of the eggs is carried out according to the "Monitoring and measuring" procedure. Each batch is examined with an ovoscope before marking and packaging.	The verification of the microbiological and physico-chemical conditions is done by collecting samples according to the self-control program and analyzing them in authorized laboratories with which the unit collaborates.
Marking and packaging	Eggs are packed in formwork, they are palletized and wrapped. Eggs are marked in an automated system with the code of the farm of origin and the expiration date. Sale of eggs in bulk: information are communicated visibly and perfectly legibly, information regarding: quality category; weight category; the way of raising chickens; manufacturer code; explanation of the meaning of the manufacturer's code; and minimum validity date. The bands and labels for category A eggs will be white, and the indications will be printed in black.	Marking of packages containing category A eggs: - the packages containing category A eggs have written on the outside, easily visible and perfectly legible; - packaging center code; the meaning of the code is explained on the outside or inside the packaging; letters and numbers of at least 2 mm; - Quality category category A or by the letter A accompanied or not by the mention "fresh"; - Weight category; a 12 mm circle around the mark for the weight class, consisting of letters at least 2 mm high; - Storage conditions "keeping eggs in the refrigerator after purchase"; - Method of raising chickens: "eggs raised in batteries"; - Minimum validity date: it must be a maximum of 28 days calculated from the laying date; letters and numbers of at least 2 mm, including the day and month; for packaging "to be consumed, preferably before … "; for the egg, the date of minimum durability followed by the date, the day, expressed in numbers from 1 to 31 and the month expressed in letters from 1 to 12 or 4 letters from the alphabet; - The "extra" mentions can only be used on packages containing category A eggs until the 9th day after laying; the laying date and the 9 days must be written; - The way of feeding the chickens can also be indicated. Tolerance regarding the marking of packaging and eggs A tolerance of 20 % is allowed for eggs bearing illegible markings during batch and packaging control.
Storage, transport, documentation	5–18^o^C, in clean spaces, free of pests. Eggs should not be refrigerated in spaces with a temperature <5 °C.	Eggs are transported with properly equipped, authorized, and well-sanitized means of transport. During transport, the cold chain must be maintained. Eggs are delivered according to the "Product release" procedure. Documents: The transport of eggs is accompanied by the following documents: shipping notice, declaration of conformity
Terms of validity	28 days from the date of laying.	
Intended use	Chicken eggs are widely used in many types of food, both sweet and salty, including baked ones. Eggs can be scrambled, fried, boiled, soft-boiled and pickled. They can also be eaten raw, although this is not recommended for people who may be particularly sensitive to salmonellosis.	The average weekly consumption of eggs should be reduced to 4 pieces. Eggs are part of the group of potentially allergenic foods, they can cause allergies.

#### 3.2.3 Flow diagram

**Fig. 1:**
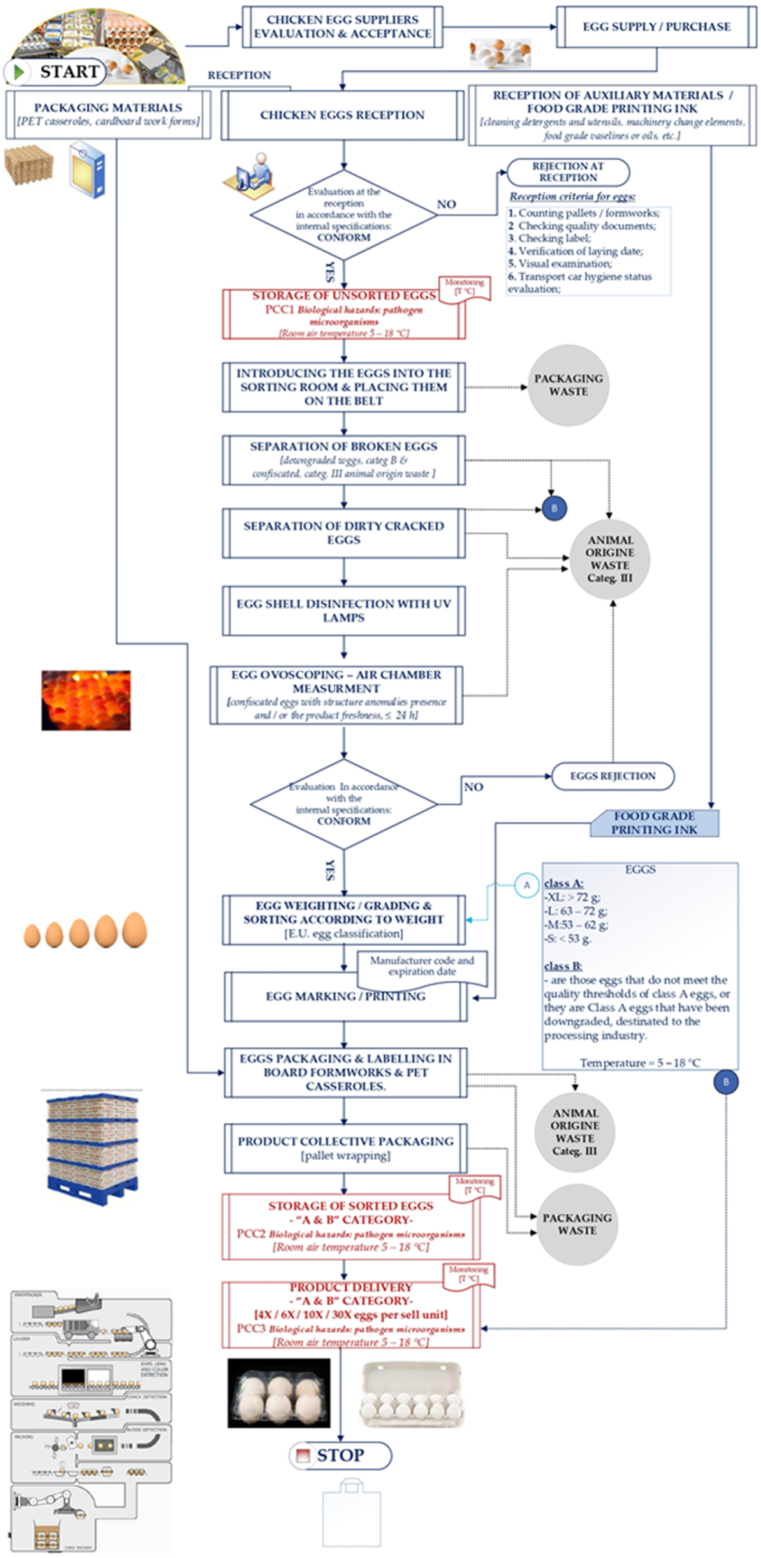
Flow diagram. (PET - Polyethylene Terephthalate; PCC - Critica Control Point; SNCU category III - food by-products of animal and non-animal origin).

The flow diagram encompasses all the stages of the technological process at the three-egg sorting and packaging units (see Fig. 1). The diagram illustrates not only the various stages of the technological process, but also includes the processes leading up to the final delivery of the product to the consumer. Providing this material is crucial to enhance the presentation of situations that may impact on the safety and security of the product. These aspects should be considered because of their significance [21]. The food safety team conducted on-site verification of the flow charts. Fig. 1 illustrates the several stages involved in the egg producing process. Each facility's reception area is designated for the collection of eggs and supplementary materials, including packaging and printing ink, where an initial examination and document verification are carried out. The unsorted egg storage area is a room with temperatures ranging from 5 to 18 °C, specifically designed for the temporary storage of unsorted eggs prior to processing. The subsequent area, the sorting room, serves as the primary place for sorting, checking for defects, and then processing the eggs. This encompasses conveyors for egg transportation, units for separating non-compliant eggs (broken or contaminated), disinfection units (UV), and ovoscopic inspection tools. In the grading and marking process, the eggs are weighed, classified, and labeled according to their size (Class A or B). The packing room comprises packing lines (automatic or semi-automated), where eggs are packaged in PET trays or cardboard containers. Also, the collection of eggs for palletization takes place in this location.

#### 3.2.4 The concepts of the HACCP plan (steps 7–12)

Assessing risks and establishing permissible thresholds. as previously mentioned, the product description of eggs can be found in detail in Table 2. The process is detailed in Fig. 1. The identification and analysis of hazards are described in Table 3. As shown in the table, the mean relative humidity ranged between 70 and 80 % across all facilities, aligning with optimal storage conditions. The following table illustrates the technological steps and potential hazards, emphasizing the importance of CCP1 (storage of unsorted eggs) and CCP3 (product delivery) for ensuring compliance. Table 4 outlines the identification and management of critical control points and control points within the analyzed egg sorting and packing units. Consequently, while CCPs concentrate on mitigating food safety risks, including physical, chemical, and biological hazards, CPs emphasize operational controls to uphold egg quality and traceability. These control points are designed to guarantee adherence to food safety requirements, product safety, and consumer trust.

**Table 3:** 

The step of the technological process	Identify potential hazards introduced, controlled, or improved at this step		Does this potential hazard need to be addressed in the HACCP plan? Yes/No	Justify your decision	Hazard assessment			What measure(s) can be applied to prevent or eliminate the hazard or reduce in to an acceptable level?
					S	P	HR	
General, for all steps	**B**	Human diseases such as the SARS-CoV-2 virus or different zoonosis	Yes	It can lead to comsumer health impact	3	1	3	- checking the employees upon entering the unit, measuring the body temperature, and observing their health status, including the presence of symptoms characteristic of the SARS CoV-2 virus; - applying disinfection and hand hygiene rules for all personnel upon entering the unit, wearing a face mask and disposable gloves; - supervision of the personnel who handle the eggs, inclusive health status verification (2x/year parazitologic, bacteriologic, and clinic general exams); - compliance with the principle "FEFO" and stock rotation; - appropriate hand hygiene, according to the Hand Washing SOP (changing min. 1x/hour of the dirty gloves for the people handling eggs or when necessary, plus washing and disinfecting hands at entering each time the production area); - wearing appropriate work equipment for protection (white for production employees and dark blue for the maintenance staff), with a minimum of three complete rows of equipment for each employee; - washing of all work equipment by an external service provider (1x/week collecting); - checking the washing efficiency for the work equipment by taking internal sanitation tests for the washed protective equipment 1 x/week (RLU reading → directly proportional to the amount of ATP collected from the sample) and externally, accreditating ISO 17025 test reports at a frequency of 2 x/year for the following parameters: aerobic total viable count (TVC) and coliforms; - restricting staff access to the areas in accordance with the job description; - personal training with the specific SOP (Standard Operational Procedures), as follows: HACCP system and CCP monitoring, personal hygiene, sanitation program, measuring and monitoring devices, production, reception, storage, allergen management, food fraud, food defense, foreign body management, and management of transport;
	**C**	Chemical residues of substances used inside the facility	No	It can lead to comsumer health impact	2	1	2-	
	**P**	Foregn bodies from company infrastructure	No	The presence of these hazards have low impact; in general lead to damages of the egg, which will not be delivered to the consumer	2	1	2-	
Egg supplier election	**B**	Presence of *Salmonella* spp. and *Campylobacter jejuni* for the supplied eggs	Yes	It can lead to comsumer health impact	3	1	3	- defining criteria for approving egg suppliers based on internal supply protocols; - writing the technical specifications and establishing acceptability criteria for eggs; - analysis of the product in accordance with established criteria (legal base); - evaluation of egg suppliers according to internal supply/purchase procedures; - training of purchase department employees in order to understand and respect the acceptability criteria; - application of the provisions of the allergen management procedure and vulnerability study; - identification of allergenic products according to Reg. E.U. 1169/2011 (at reception, if that is the case); - re-evaluation of suppliers where non-conformities were identified at the reception; - removing from the list of accepted suppliers those who do not meet the acceptance conditions established by the supply procedure after complaint management and recurrence of the same issue;
	**C**	Pesticide residues, mycotoxins, heavy metals, drugs, hormones, dioxines, radioactivity, allergens (other than eggs protein).	Yes	It can lead to comsumer health impact	3	1	3	
	**P**	Presence of insects, rodent droplets, plastic, glass.	No	The presence of these hazards have low impact; in general lead to damages of the egg, which will not be delivered to the consumer	2	1	2	
	Fraud		No	97 % of the eggs come from own farms; exception → unit A with 5 external suppliers, and the matrix does not lead itself to fraud	2	1	2	
Egg supply	**B**	Development of pathogenic microorganisms due to improper transport temperature *(Salmonella* spp. and *Campylobacter jejuni).*	Yes	It can lead to comsumer health impact	3	1	3	- supply only from evaluated and accepted suppliers, which have been previously evaluated and signed the quality and food safety annex related to the cold chain maintenance; - the transport of eggs with isothermal vehicles, authorized sanitary-veterinary, properly sanitized; - training of employees from the reception to check elements of compliance related to transport temperature, hygiene, and egg quality conditions;
	**C**	Chemical residues of substances used to sanitize means of transport.	Yes	It can lead to comsumer health impact	2	1	2	
	**P**	Contamination with foreign bodies during transport: minerals, insects parts, rodents, dust	No	The presence of these hazards has a low impact; in general, they lead to damage to the egg, which cannot be processed further or delivered to the consumer.	2	1	2	
Reception	**B**	Development of pathogenic microorganisms due to farm conditions and/or improper transport: *Salmonella* spp.*, Campylobacter jejuni;*	Yes	It can lead to comsumer health impact	3	1	3	- reception of egg batches on an arranged and boulded ramp (with temperature control); - reception of eggs in accordance with the established criteria [f.e., 1 x/month externally accredited ISO 17025 test reports for the biological hazards *Salmonella* spp. and *Campylobacter jejuni*]; - training of employees from the reception to check elements of compliance (transport temperature inside the vehicle and sanitization status, correctness and legibility of the inscriptions on the quality documents and/or label applied to the pallets, and the declaration of conformity, shelf life of the product);
	**C**	Pesticide residues, mycotoxins, heavy metals, melamine, drugs, hormones, dioxins, radioactivity, allergens (eggs protein), chemical residues of substances used to sanitize farms and means of transport;	Yes	It can lead to comsumer health impact	3	1	3	- receipt of eggs in accordance with the established criteria [for example, 1x/year carried out accredited ISO 17025 test reports from each egg supplier for the chemical hazards: mycotoxins, heavy metals, drugs, hormones, dioxins, radioactivity, allergens (other than egg protein), chemical residues of substances used to sanitize the farms (e.g., fipronil) or means of transport, and once per year, the same parameters measured and assessed by the company in accordance with the autocontrol program]; - rejection at the reception of egg batches for the following reasons: improper temperature, improper status of truck hygiene; - rejection at the reception of the suspicious eggs that exceed the first 1/3 of the shelf life (eggs ≥10 days from lying), eggs that present sensory parameters changed and are contaminated with pest signs;
	**P**	Presence of minerals, insects, rodents, rodent drops, plastic, glass, metals, etc. Contamination with foreign bodies during transport:	No	It can lead to comsumer health impact	2	1	2	- compliance with the monthly disinsection and deratization program in the outside yard and reception ramp; - protected lights on the ramp and compliance with the annual maintenance of burdock loading (functionality and integrity); - training of the internal person responsible for pest control activities, the person who maintains the relationship with the service provider; - monitoring of the plastic elements from the burdock unloading ramp;
	**-**	Food fraud	No	97 % of the eggs come from own farms; exception → unit A with 5 external suppliers, and the matrix does not lead itself to fraud	2	1	2	- rejection at the reception of egg batches without provenance documents whose origin cannot be traced; - application of the provisions of the vulnerability study; - reevaluation of the supplier and decision-making about keeping it or closing collaboration with it in case of fraud;
	**-**	Food Defence	No	It can lead to comsumer health impact	2	1	2	- application of the Food Defense Plan as a result of the assessment: 24/7 surveillance cameras, controlled access inside the site yard, control access systems for employees according to job descriptions, and mitigation strategies related to employee release;
Reception of packaging [cardboard packaging, paper rolls, PET casserols, PP bags], labels, and food-grade ink	**B**	Presence of *Total viable count* (TVC) and *coliforms*	Yes	It can lead to comsumer health impact	3	1	3	- supplying only from accepted suppliers, GFSI-certified (Global Food Safety Initiative); - completing the supply order with quality and food safety requirements (microbiological parameters); - transport carried out with properly sanitized vehicles; - internal sanitation test at each reception (RLU) and external accreditated test reports min. 1x/year for microbiological criteria specified by Romanian Ministry of Health Order No. 976/1998 [1] and Regulation (EC) No. 1935/2004 [2];
	**C**	Components that can migrate into the product (global migration, heavy metals), toxic substances in the marking ink	Yes	It can lead to comsumer health impact	3	1	3	- checking the supplied products and accepting only those that meet the quality and food safety requirements, accompanied by appropriate documents. → for packaging: Declaration of Conformity at each delivery; Compliance Declaration; Migration Test Reports (global migration of the components, organoleptic modifications, specific heavy metal migration); and Technical Data Sheet (min. 1x/year); → for ink: use of food-grade ink; acceptance criteria as described in Reg. (EC) No. 2006/2023 [3] regarding the good manufacturing practices for materials and articles intended to come into contact with food, and Reg. (EC) No. 1935/2004, art. 3; - rejection at the reception in case of non-conform packaging and/or ink; - compliance with the reception SOP;
	**P**	Presence of metals, glass, dust, insects, rodents traces	No	It can lead to comsumer health impact	2	1	2	- protection of lighting sources from the outside ramp; - checking the integrity of transport packaging; - checking the presence of traces of insects or rodents inside the vehicle;
Storage of unsorted eggs	**B**	Proliferation of pathogenic microorganisms in favorable temperature conditions results in the formation of condensation on the eggshell. Contamination from the storage space *Salmonella* spp, *Campylobacter jejuni, TVC, Moulds)*	Yes	It can result an unsuitable product or possibly have a health repercussion leading to various illnesses.	3	1	3	- stablishment of storage conditions: temp. of 5–18 °C, and temperature and storage time monitoring; - appropriate sanitation of the storage warehouse, in accordance with GMP and GHP; - metrological checks at least annually of measuring and control devices (externally) and monthly (internally with calibrated standard equipment), plus annual checks of the warehouse storage climate unit; - enhancing the efficiency of refrigeration systems by preventing excessive load on the storage warehouse; - personal training with the storage SOP (standard operational procedure) and sanitation of the warehouse SOP; - externally accredited ISO 17025 sanitation tests (1x/3 months) for surfaces [from storage rakes, around sinks, corners, and under the cooling system] and for the air; - application of batch sheets and verification of shelf life;
	**C**	Residues from pest control activities and/or cleaning chemicals	No	The presence of this hazards can cause illness and injury to the consumer	2	1	2	- compliance with the monthly disinsection and deratization program of the company yard and reception ramp (chemicals used, concentrations, frequency); - compliance with the internal annual sanitation program; - training by the service provider of the internal responsible for pest control activities; - prohibition of storing products or other substances than eggs in the storage warehouse;
	**P**	Cracked egg. Contamination with foreign bodies during storage and internal manipulation from the storage: glas, parts of insects, hard plastic, dust	No	The presence of these hazards has a low impact; in general, they lead to damage to the egg, which cannot be processed further or delivered to the consumer.	2	1	2	- training of the responsible staff with the Foreign Body Management SOP; - monitoring the integrity of lighting, hard plastic transport egg forms, hard plastic electrical systems, and plastic pallets; - monitoring of all entries and openings (completely sealed doors, good nets at windows); - presence of EFK (electro-fly killers with glue tape) at all entries;
Storage of packaging	**B**	Contamination from the storage space (*TVC, Moulds*)	No	It can lead to comsumer health impact	2	1	2	- sanitization of storage warehouses according to a daily, monthly, and annual sanitization program; - complying with storage rules: on shelves, pallets with the batch label; the packaging is stored in the warehouse in designated areas that are specifically marked and separated between different types of packaging; - personal training with the requirements of the packaging storage SOP (ambiental temperature and RH max. 40 %); - compliance with the „FIFO” principle;
	**C**	Residues from pest control activities and/or clenning chemicals	No	The presence of this hazards can cause illness and injury to the consumer	2	1	2	- compliance with the monthly disinsection and deratization program of the storage area (chemicals used, concentrations, frequency); - compliance with the internal annual sanitation program; - training by the service provider of the internal responsible for pest control activities; - prohibition of storing products or substances other than packaging materials;
	**P**	Presence of glass, insects, rodents, dust	No	It can lead to comsumer health impact	2	1	2	- keeping the packaging protected against dust contamination and other foreign bodies by ensuring the integrity of the protective packaging; - checking the integrity of individual packaging during storage, at least once a week, and eliminating non-compliant packaging: deformed, dusty, broken, or contaminated with foreign bodies; - applying a distance of 50 cm free from the wall for all pallets; - compliance with the monthly disinsection and deratization program in the storage warehouse; - protection of lighting installations; - monitoring of all entries and openings (completely sealed doors, good nets at windows); - transparent foil appliance on all windows; - all entrances to the exterior are protected against the penetration of insects (presence of EFK: electro-fly killers at all entries) and pests (internal rodent traps); - training of the responsible staff with the Foreign Body Management SOP; - restricting staff access to the storage space;
Introducing eggs for sorting	**B**	Contamination from personnel, MOBA work line or working space (*TVC, Moulds,* *Staphylococcus haemolyticus,* *Staphylococcus coagulase positive,* *Enterobacteriacea)*	No	It can lead to comsumer health impact	2	1	2	- personal health status check at a daily frequency at the pre-operational control (drawn up by the occupational medicine doctor); - proper handling of the eggs to prevent breakage; - removing the packaging film used for the protection of the pallets without making contact with the eggs and removing them from the sorting room; - establish and follow the MOBA packaging line sanitization program (concentrations, operating time, frequency) according to the GHP and producer guidelines from the technical book; - daily check of the ventilation system to prevent condensation and its removal in case of appearance; - personal training with the egg sorting SOP; - weekly internal sanitation tests for surfaces (RLU); - biannually, the company makes externally accredited ISO 17025 test outcomes for operational surfaces, individuals' health conditions, and working microaeroflora.
	**C**	Residues from pest control activities and/or clenning chemicals	No	The presence of this hazards can cause illness and injury to the consumer	2	1	2	- compliance with monthly disinsection and deratization program of thesorting area (chemicals used, concentrations, frequency); - compliance with the internal annual sanitation program; - training by the service provider of the internal responsible for pest control activities; - prohibition of storing products or substances other than eggs; - application of the provisions of the Allergen Management Procedure for the people: lunch area; - uses of food grade vaselines for greasing equipment (NSF);
	**P**	Presence of glass, metal, insects, rodents or rodents traces	No	It can lead to comsumer health impact	2	1	2	- collection of animal and non-animal waste in labeled containers and their removal from the sorting area at the end of the program; - sorting the eggs, removing the cracked, broken, and dirty ones: dirty eggs are placed on the formwork, and broken eggs are collected in containers provided with PP or PET bags, which, after filling, will be tied to the mouth and then stored in the refrigerated space [temp. 5–18 °C] for non-compliant products, with a view to delivery for neutralization or further industrial processing – external plant;
Separation and removal of confiscated, dirty and cracked eggs	**B**	Contamination from machinery, and personnel; Contamination due to breaking eggs	No	It can lead to comsumer health impact	2	1	2	- ensuring and recording the temperature in the sorting room [temp. of 10–12 °C for max. 5 h]; - broken eggs are collected in containers provided with PP or PET bags, which, after filling, will be tied to the mouth and then stored in the refrigerated space [temp. 5–18 °C] for non-compliant products, with a view to delivery for neutralization or further industrial processing at an external plant; - use of clean, single-use formwork; - identification of sorted eggs by the label corresponding to each category [A and B]; - preparation and compliance with the MOBA equipment and installation maintenance program;
	**C**	Contamination with oils used to lubricate equipment. Contamination with residues and substances used for sanitation and pest control activities	No	the presence of residues of oils used for greasing or washing substances cannot cause serious illness	2	1	2	- the use of food grade lubricating oils; - personal training with the Egg sorting, marking and packaging SOP; - for MOBA parts in contact with the products, the company use only food grade vaselines for greasing equipment (NSF);
	**P**	Presence of glass, metal, parts from other eggs	No	It can lead to comsumer health impact	1	1	2	- monitoring of the semifinished products (sorted, unpacked eggs, clean and conform as shape and structure); - monitoring the integrity of MOBA line elements and of the production environment;
Disinfection with UV lamp	**B**	Inefficient disinfection [for Total viable count of germs (TVC), coliform bacteria]	No	It can lead to comsumer health impact	2	1	2	- preparation and compliance with the UV lamp maintenance program in accordance with the manufacturer's technical data sheet; - checking the operation efficiency through external test reports (min. 1 × 3 months); - replacement of lamps after 10,000 h of functioning (aprox. 1 year);
	**C**	**-**	–	–	–	–	–	–
	**P**	Presence of: glass, hard plastic, metal	No	It can lead to comsumer health impact	2	1	2	- daily check of the UV integrity lamp (before and after batch finishing);
Egg ovoscopy and air chamber measurement	**B**	Inappropriate removal of eggs with dirty shell, broken	No	It can lead to comsumer health impact	2	1	2	- daily check of ovoscopy operation: eggs whose air chamber is movable and/or exceeds 6 mm in height are removed, classified as category B eggs, and stored in formwork together with cracked and dirty eggs, which further will be delivered to industrial enterprises for processing; - use of clean, single-use formwork; - identification of sorted eggs by labeling corresponding to each category; - exercise caution when handling the eggs to prevent any inadvertent breakage; - compliance with the autocontrol program for sanitation tests in the working flow;
	**C**	Contamination with residues and substances used for sanitation and pest control activities or oils used to lubricate equipment.	No	the presence of residues of oils used for greasing or washing substances cannot cause serious illness	2	1	2	- the use of food-grade lubricating oils; - personal training with the Egg Ovoscopy SOP; - for ovoscope parts in contact with the products, the company uses only food-grade vaselines for greasing equipment (NSF); - complying with the ovoscope maintenance program; - compliance with the monthly disinsection and deratization program of the company yard and reception ramp (chemicals used, concentrations, frequency); - compliance with the internal annual sanitation program;
	**P**	Presence of metals, glass, plastic	No	It can lead to comsumer health impact	2	1	2	- preparation and compliance with the maintenance program for utilities and installations; - ensuring and recording the integrity of ovoscope and of equipment lighting system;
Weighing eggs and sorting according to weight	**B**	Development of pathogenic bacteria in favorable temperature conditions. The formation of condensation on the egg shell. Contamination from machinery or working area.	Yes	It can lead to comsumer health impact	3	1	3	- ensuring the maintenance of the cold chain and recording the temperature in the sorting room to prevent the formation of condensation on the egg shell; - monitoring of the temperature and documenting the information inthe file working sheet, inclusive the packing report with information about the lot no. of each packagin used; - checking the weighing and sorting processes according to 4 categories applied: XL, L, M, S; - checking the weighing and sorting machine with standard calibrated weights, minimum 1 x/month; - completing the egg weight check register and tracking of mass balance; - internally performed sanitation tests for the working surfaces (RLU expresed) are done every month, and externally accredited ISO 17025 sanitation tests are performed every three months for surfaces, including weighted lines or air; - reception of eggs in accordance with the established criteria [f.e., 1 x/month externally accredited ISO 17025 test reports for the biological hazards *Salmonella* spp. and *Campylobacter jejuni*];
	**C**	Residues of chemical substances used for sanitation of MOBA equipments; lubricants	No	It can lead to comsumer health impact	2	1	2	- establishing and following the Sanitation Program (concentrations, action time, frequency) according to the internal sanitation equipment (MOBA) program; - internal pH test of the rinse potable water taken from the cleaned line (conform rinse potable water pH between 6.5 and 9.5);
	**P**	–	–	–	–	–	–	–
Marking eggs/Printing	**B**	**-**	–	–	–	–	–	
	**C**	Heavy metals in the substances used for marking/printing [Pb, Cd, As, Hg)	No	It can lead to comsumer health impact	2	1	2	- checking the automatic egg stamping correctness and legibility of marking and shelf life; - supplier compliance declaration for the ink used; - accreditated ISO 17025 test reports for heavy metals presence in eggs [min. 1 x/year] and from the supplier [in ink]; - compliance with the maintenance program of the MOBA printing part;
	**P**	Presence of glass, metal, insects, rodents	No	It can lead to comsumer health impact	2	1	2	- personal training with the SOP for foreign bodies monitoring related equipment [f.e., displaies, hard plastic from carcasses, conveyor belts, etc.]; - monitoring the integrity of marking and printing MOBA parts equipment; - visual inspection; - compliance with the monthly disinsection and deratization program of the company yard and reception ramp (chemicals used, concentrations, frequency);
Egg packaging in formwork and labeling	**B**	The development of pathogenic bacteria in favorable temperature conditions. The formation of condensation on the surface of the eggs. Contamination from packaging materials.	Yes	It can lead to comsumer health impact	3	1	3	- using approved single-use packaging for the food industry; - checking the microbiological load of packaging through sanitation tests (internal sanitation tests, RLU) and externally accredited ISO 17025 sanitation tests (TVC and Coliforms); - maintenance of the temperature of the sorting and packaging area between 5 and 18 °C, monitoring it and documenting it in the temperature sheet file: for avoidance of condensation on the egg shell; - checking the cleaning status of formwork, casseroles, and pallets for egg class A: weights S, M, L, and XL;
	**C**	Chemical components that can migrate from the packaging to the product. Residues from substances used for sanitation and pest control activities.	No	It can lead to comsumer health impact	2	1	2	- annually migrations test for all packagings used from the suppliers (global migration of the components, organoleptic modifications, specific heavy metal migration); - uses only of food garde vaselines/lubricants (NSF); - compliance with the monthly disinsection and deratization program from the packaging area; - compliance with the internal annual sanitation program;
	**P**	Presence of glass, metal, insects.	No	It can lead to comsumer health impact	2	1	2	- visual inspection of packaged products; - compliance with the maintenance program of MOBA equipment; - proper intermediary storage of eggs during the working shift, handling, collection of packaging, and correct disposal of packaging waste; - avoidance of prolonged storage of packaged products; - monitoring the integrity of hard plastic objects of the line and from the packaging area; - compliance with pest control activities;
Storage of sorted eggs, category A and B	**B**	Development of pathogenic bacteria, due to improper storage conditions (*Salmonella* spp.*, Campylobacter jejun*, Aerobic TVC, Moulds, Coliforms)	Yes	it can lead to obtaining an inappropriate product or even to a health impact causing different diseases.	3	1	3	- establishment of storage conditions: temperature of 5–18 °C, monitoring it, and documenting it in the temperature sheet file; - precooling the warehouse before introducing and storing the eggs; - avoidance of placing pallets in front of the air cooling system; - observing the formation of condensation, its removal, and the backup movement of the final product in the second warehouse while the company makes a backup check of the cooling system; - compliance with the annual disinfection program for the warehouse spaces; - checking the microbiological load of surfaces through sanitation tests 1 x/week (internal sanitation tests RLU) and external accreditated ISO 17025 sanitation tests 2 x/year: warehouse microaeroflora (*Aerobic TVC, Moulds*) and 1 x/trimester for surfaces (*Aerobic TVC, Coliforms*);
	**C**	Residues of chemicals from cleaning operations and/or pest control activities	Yes	It can lead to comsumer health impact	2	1	2	- compliance with the sanitation program for spaces and with the monthly pest control program (chemicals, concentrations, frequency); - checking the efficiency of rinsing after cleaning, pH of rinse water, sample taken from the warehouse walls during drying time (conform rinse potable water pH between 6.5 and 9.5); - training of the employees responsible for sanitation activities at the final product warehouse storage facility;
	**P**	Presence of glass, plastic, insects, rodents	No	It can lead to comsumer health impact	2	1	2	- training of the employees with the Foreigh Bodies management SOP; - monitoring the integrity of equipment; - transparent foil to all windows; - the sorting plant strictly prohibits the use of hard plastic (exceptions: MOBA component parts) or glass utensils, only allowing the use of bendable plastic;
Product delivery A and B category	**B**	Development of pathogenic bacteria as a result of non-compliance with storage temperatures or the formation of condensation on the surface of the eggshell (*Salmonella* spp.*, Campylobacter jejuni,* Aerobic TVC, Moulds, Coliforms)	Yes	It can lead to obtaining an inappropriate product or even to a health impact causing different diseases.	3	1	3	- checking the state of hygiene of the means of transport; - checking the temperature in the truck transport room; - temperature monitoring during transport – visual inside driver cabin, thermogram printing at the end of the journey, and 1x/year-blind verification of the transport service providers through dataloger insertion inside the pallet with the eggs (during the summer period); - loading for delivery at the appropriately arranged ramp or burdock loading ramps, sanitized according to the Cleaning annual program; - before loading, checking the integrity of the packaging and the product shelf life; - drivers with up-to date checks for health status; - conducting the transportation using vehicles that have been authorized by the Food Safety Authority and that are able to maintain the required temperatures throughout the whole journey;
	**C**	Residues of chemicals from cleaning operations, fuel residue, or other residue from products transported	No	It can lead to comsumer health impact	2	1	2	- conducting the transportation using vehicles that are properly cleaned, with no smells; - the transportation exclusively contains eggs and does not involve the movement of other goods utilizing a coupling mechanism;
	**P**	Presence of impurities: metal, plastic protection lamps, and stitches from windows	No	It can lead to comsumer health impact	2	1	2	- verification and monitoring of the hygiene and integrity of means of transport; - careful handling of products to preserve the integrity of protective and transport packaging; - verification of the technical condition of the means of transport; - check inside the truck to not be transported other than eggs;

1. 1998, O.n.d.d., *ORDIN nr. 976 din 16 decembrie 1998*.

Romanian Ministry of Health 1998.

2. COUNCIL, R.E.N.O.T.E.P.A.O.T. and o.O. 2004, *REGULATION (EC) No 1935/2004 OF THE EUROPEAN PARLIAMENT AND OF THE COUNCIL*.

*of October 27, 2004.* THE EUROPEAN PARLIAMENT AND OF THE COUNCIL, 2004.

3. 2023/2006, C.R.E.N. and o.D. 2006, *COMMISSION REGULATION (EC) No 2023/2006 of December 22, 2006.* Official Journal of the European Union, 2006.

**Table 4:** CCP/CP identification.

Process step	Significant hazard	Q1^1^	Q2^2^	Q3^3^	Q4^4^	CCP/CP YES/NO
Egg supplier election	**B** [f. e.: *Salmonella* spp. and *Campylobacter jejuni*]: the supplied eggs	Yes	No	No	–	**CP 1**
	**C** [f.e.: pesticide residues, mycotoxins, heavy metals, drugs, hormones, dioxins, radioactivity, allergens (other than eggs protein).]: eggs can be contaminated from the farm;	Yes	No	No	–	
Egg supply	**B** [f.e.: *Salmonella* spp. and *Campylobacter jejuni*]: eggs can be contaminated from improper transport temperature;	Yes	No	No	–	**CP2**
Reception	**B** [f.e.: *Salmonella* spp, *Campylobacter jejuni, TVC, Moulds*]: egg supplier election	Yes	No	No	–	**CP 3**
	**C** [f.e. Pesticide residues, mycotoxins, heavy metals, melamine, drugs, hormones, dioxins, radioactivity, allergens (eggs protein), chemical residues of substances used to sanitize farms and means of transport]: egg supplier election	Yes	No	No	–	
Reception of packaging materials, labels and ink	B [f.e. TVC, coliforms]: contamination from the manufacturer or transport;	Yes	No	No	–	**CP 4**
	**C** [chemicals residue, overall migration limit (OML) for plastic packaging >60 mg/kg food, or 10 mg/dm^2^ of the contact material]: contamination from the manufacture;	Yes	No	No	–	
Storage of unsorted eggs	**B** [f.e.: *Salmonella* spp]: contamination due to the improper temperature [limits → 5–18 °C];	Yes	No	Yes	No	**CCP - 1**
Weighing eggs and sorting according to weight	**B** [f.e.: *Salmonella* spp.*, Campylobacter jejuni, TVC, Coliforms*]: contamination of eggs due to improper temperatures, condensation or equipment;	Yes	No	No	–	**CP - 5**
Egg packaging in formwork and labeling	**B** [f.e.: *Salmonella* spp.*, Campylobacter jejun, TVC, Coliforms*]: contamination of eggs due to improper temperatures, condensation or equipment;	Yes	No	No	–	**CP - 6**
Storage of sorted eggs, category A și B	**B** [f.e.: *Salmonella* spp.*, Campylobacter jejun*, Aerobic TVC, Moulds, Coliforms]: contamination due to the improper temperature [limits → 5–18 °C];	Yes	No	Yes	No	**CCP - 2**
Product delivery A and B category	**B** [f.e.: *Salmonella* spp.*, Campylobacter jejun*, Aerobic TVC, Moulds, Coliforms]: contamination due to the improper temperature [limits → 5–18 °C];	Yes	No	Yes	No	**CCP - 3**

The initial critical control point (CCP) identified pertained to the storage of unsorted eggs. Failure to adhere to the specified settings during this stage may result in the proliferation of harmful bacteria, hence posing potential health risks to customers.

The second CCP is designated for the storage of categorized eggs, specifically those classified as category A and B. The current temperature in the technical process ranges from 5 to 18 °C. Elevated temperatures can result in an escalation of the microbial burden, or temperature variations that result in condensation and subsequently toxins. Strict regulations of time and temperature can effectively manage bacterial development. Hence, it is imperative to meticulously monitor both the duration and the degree of heat during the storage procedure. The distribution and sale stages must adhere to the same stringent requirements, as specified by CCP 3 [9,13].

Table 5 provides detailed information on the critical limits, monitoring methods, and necessary actions to be conducted if the critical limits or action limits or action criteria are exceeded, following the successful execution of the CCPs. Critical limits for storage conditions (5–18 °C) were strictly monitored. Deviations exceeding 18 °C for more than 3 h required immediate corrective actions, such as transferring eggs to alternative storage (Table 5). These measures ensured microbial growth were controlled effectively.

**Table 5:** Identifying critical limits, monitoring procedures, and corrective actions.

CCPs	Significant hazard (s)	CCP parame-ter	Value programmed and validated	Critical limits	Monitoring procedure				Correction and Corrective action	Records
					What?	How?	When-frequenc y?	Who?		
**Storage of unsorted eggs** **CCP – 1** **Storage of sorted eggs, category A and B** **CCP - 2**	**Biological hazard**: Proliferation of pathogenic microorganisms in favorable temperature conditions, formation of condensation on the eggshell; Contamination from the storage space.	Temp. in the storage room	5–18 °C	>18^o^C for more than 3 h	Air temp.	Reading and recording storage space temperature Checking/validating the internal system with the ethalon thermometer [standard measuring and monitoring devices].	Continue through electronic systems and physical by the stockkeeper 2 x/day from Monday to Sunday.	Monitoring: the stockkeeper and the person responsible for security during the weekend; Verification: Quality Assurance Manager and Production Responsible; Corrective action: Production Responsible and/or Administrator;	**Correction** If the temperature is near the critical limit (>15 °C), immediate notification of the technical department and production responsible is done. During storage, a free space is ensured between the formwork and boxes, sufficient for cold air circulation. If the defect cannot be fixed and there is a danger that the temperature of the egg warehouse [at reception or at delivery] will exceed the value of 18 °C, the eggs should be urgently inserted for sorting if possible or transferred to another space with a corresponding temperature of 5–18 °C [case of CCP1], and/or delivered immediately or transferred to another space with a corresponding temperature of 5–18 °C [case of CCP2]. If the temperature of the air warehouse has reached >18 °C for more than 3 h, the product lots are identified as potentially unsafe and treated according to the procedure "Control of non-compliant products [quarantified, externally tested reports for Salmonella and sensory parameters, and the decision of the Food Safety Team]. Sorting, packaging, and commercialization within a maximum of 3 days of eggs that have been stored at a temperature <5 °C. Moldy, rotten, cloudy, or even opaque eggs, without separation between white and yolk, or those with dark spots on the inner side of the shell, produced by various moulds or bacteria, are confiscated and destined for denature. **Corrective action** Maintaining the annual verifications of the cooling system according to the internal schedule for preventive measures. If the electricity supply stops, the electric group will be automatically turned on to ensure the appropriate conditions. Establishment and application of equipment maintenance program Establishing and following the specific training of the employees (on food safety and on technical part).	Online system database and temperature sheet
**Product delivery** **CCP - 3**	**Biological hazard**: Development of pathogenic bacteria as a result of non-compliance with storage temperatures, or the formation of condensation on the surface of the eggshell; contamination from the means of transport.	Temp. during product delivery [inside the truck]	5–18^o^C	>18^o^C for more than 3 h	Air temp.	Reading and recording the temperature inside the truck at product loading; Automatic system, checking the thermodiagrame before unloading the product	Continue through electronic systems. Visualisation every 2 h during transport [inside the driver cabine]	Monitoring: the driver Verification: Logistic Responsible Corective action Logistic Responsible and/or Administrator	**Correction** The product is not loaded in the truck until the temperature of the truck-transported room is max. 10 °C. In case of failure of the system, the truck will be changed (maximum 3 h) or will be redirected nearest the closest refrigerated warehouse [due to our networking partners and collaborations]. **Corrective action** Revision in time on all trucks and on all refrigerated systems. between If the temperature are not in the range 5–18^o^C Compliance with GMP, GHP measures and staff training. Respecting the product legal parameteres and compliance with product technical parameters; Corect sanitation of the transport trucks after easch delivery [thawing process and sanitation];	Thermodiagram picture at delivery

To assess the effectiveness of the HACCP plan, the food safety team devised a verification plan in Table 6. This plan outlines the scope, frequency, and assigned duties for the verification activities.

**Table 6:** Establishing verification procedures.

No. crt.	Field of verification/item	Frequency	Responsible for verification
1.	Verification of compliance with the procedure for selecting suppliers;	Annual or at the introduction of a new supplier in the system	Purchase Responsible
2.	Checking the quality and safety of eggs: - quality parameters (pH, sensory) → once every 3 months; - safety parameters: veterinary residue, mycotoxins, PCB, heavy metals, drugs, hormones, dioxins, melamine, radioactivity, allergens (other than egg protein), chemical residues of substances used to sanitize the farms (e.g., fipronil) → annual. - *Salmonella* spp.*, Campylobacter jejuni*: Monthly	Annual, biannual, and/or monthly	HACCP team leader
3.	Checking the conformity of transport at reception (daily or each reception) and at delivery (each delivery);	Daily or each reception/each transport	Stockkeeper Logistic responsible
4.	Checking the temperature and hygiene conditions from storage warehouses and transport until sale;	Daily/as long the product is kept in storage or transported	Logistic Responsible Production Responsible Stockkeeper Driver
5.	Potable water supply check	Annual	Hygiene Responsible
6.	Verification of compliance with the stages of the technological flow	Monthly	Technological engineer
7.	Verification of compliance with equipment maintenance	Annual, biannual, and/or monthly	Maintenance manager
8.	Verification of calibration of measuring and control device	Annual or when it is necessary.	Maintenance responsible
9.	Checking the hygiene of production protective equipment, spaces, annexes, and social groups	Internal (weekly) External (1x/3 months)	Hygiene Responsible HACCP Team leader
10.	Checking the control of the health of the staff	Biannual	Production Responsible
11.	Checking the hygiene of the work equipment	Internal (weekly) External (1x/3 months)	Hygiene Responsible HACCP Team leader
12.	Checking efficiency for waste disposal	Monthly	HACCP team leader
13.	Verification of compliance with the pest control procedure	Monthly	Hygiene Responsible
14.	Verification of CCP records; deviations from critical limits; execution of corrections and/or corrective actions	Daily	HACCP team leader
15.	Checking CP records	Daily	Production responsible HACCP team leader
15.	Checking the efficiency of employee training	Once every three months	HR Manager Production Responsible HACCP team leader
16.	Checking the quality control and safety of the finished eggs	Internal (daily) External (monthly)	Production Responsible HACCP team leader
17.	Checking the registration activity	Monthly	HACCP team secretary
18.	Checking the registration and settlement mode of complaints, trend analysis conclusions	Monthly	HACCP team leader
19.	Checking team biovigilance	Annual	TACCP team
20.	Checking the fraud vulnerability	Annual	VACCP team

The research utilizes the documents and records generated throughout the execution of the plan to fulfill the final principle of the HACCP plan. These records serve as proof of the use of HACCP principles, surveillance of CCP parameters, and suggested remedial measures. The materials are categorized into instructions and processes and are comprised of evidence-based documents. Their components consist of a title, purpose, application/scope, definitions, abbreviations, authority, duties, description of operations, records, linked papers, references, and annexes.

## 4 Findings and discussions on the analysis

### 4.1 Evaluation of quality parameters

Concerning the assessment of the quality criteria of eggs from farms A, B, and C, by Regulation (EU) 589/2008 [22], the following table emphasizes that most of the examined eggs were categorized as high quality and safe for human consumption. Thus, less than 1 % of all units were found to have eggs with abnormal shapes, indicating that most of the eggs have a typical shape. The percentage of dirty eggs exhibited a slightly elevated number, particularly in unit C, indicating that while the majority of eggs are clean, there is still a need for improvement in the handling procedures to mitigate the incidence of dirty eggs. The studied units reported a percentage of damaged eggs below 2 %, which complies with the regulations requiring eggs to be free from dirt and damage.

A small proportion of the eggs had an air cell height that was beyond 6 mm, adhering thus to the requirement that the air cell height should not exceed 6 mm. The occurrence of yolk abnormalities was not significant, in unit A having a higher frequency than unit B and C. However, all units had an incidence below 0.2 %, suggesting that the majority of yolks had no signs of significant abnormalities. Cloudiness or lack of transparency was rarely observed in the egg whites from unit A, occurring in less than 0.1 % of the cases. This indicates that nearly all the eggs had clear and transparent whites.

According to Table 7, there were no detected (nd) cases of germ growth or the presence of foreign matter, which complies with the accepted standards. Unit B experienced experienced two cases of foreign smells that resulted in the rejection of eggs at reception. This fact also suggests the implementation of efficient quality control procedures to identify and eliminate eggs that do not match the requirements established by European laws. Based on the research, the data shows a significant level of compliance with EU regulations, while there are certain places where improvements in the technological flow might be implemented. Unit C showed higher levels of dirty eggs (1.98 %), indicating potential procedural gaps in handling. However, across all units, parameters such as air cell height and microbial load remained compliant with EU standards, suggesting robust control measures.

**Table 7:** Evaluation of quality parameters.

Egg quality parameters		Unit A	Unit B	Unit C	Reference description [Reg. E.U. 589/2008]
(a) shell and cuticle:	irregular shape (%)	0.31	0.26	0.16	normal shape, clean and undamaged;
	dirty (%)	0.81	1.15	1.98	
	damaged (%)	1.49	1.53	1.27	
(b) air space:	height >6 mm (%)	0.37	0.44	0.28	height not exceeding 6 mm, stationary; however, for eggs to be marketed as ‘extra’, it may not exceed 4 mm;
	for extra eggs: height ≤4 mm (%)	nd	nd	nd	
(c) yolk:	abnormalities present at the yolk	0.16	<0.1	<0.1	reference values: visible on candling as a shadow only, without clearly discernible outline, slightly mobile upon turning the egg, and returning to a central position;
(d) white:	unclear, nontranslucent	<0.1			clear, translucent;
	development	nd	nd	nd	imperceptible development;
(f) foreign matter	presence	nd	nd	nd	not permissible
(g) foreign smell	presence	nd	presence 2 cases/rejection at reception	nd	not permissible

### 4.2 Evaluation of veterinary drugs

The following data shows that after conducting analyses on the egg sorting units, no antibiotic residues were found in eggs from units A, B, and C for chlortetracycline, erythromycin, oxytetracycline, tetracycline, and tylosin. The absence of antibiotic detection in the examined eggs indicates that their levels are below the detectable threshold of the used testing procedures, confirming their compliance with the maximum residue limits (MRLs) set by Regulation (EU) 37/2010 [18]. Regarding Neomycin, and Tiamulin, the designation "not applicable" (na) indicates that the determinations for these antibiotics were not relevant in relation to the waiting period of 0 days. However, it should be noted that these antibiotics do have established Maximum Residue Limits (MRLs). The current Maximum Residue Limit (MRL) is implemented for egg-laying poultry farms through national-level monitoring programs. Thus, the responsibility of the farmer is to conduct tests to confirm the clearance of antibiotics from the body. The lack of detectable residues in the samples amples examined indicates that the egg sorting units guarantee the safety of the eggs for human consumption in relation to the specified antibiotics. The reference to withdrawal periods for specific antibiotics underscores the significance of practicing responsible usage of veterinary drugs in poultry farming. In this context, a specific time frame must pass between the final administration of the antibiotic and the collection of eggs intended for human consumption. This precaution is taken to guarantee that antibiotic residues remain below the permissible threshold (Table 8). As seen in Table 8, no residues of veterinary drugs such as chlortetracycline or tetracycline were detected, confirming adherence to EU standards.

**Table 8:** Evaluation of veterinary drugs.

Veterinary drugs	Unit A	Unit B	Unit C	Reference values [Reg. E.U.37/2010, max. μg/kg]	Observations
	X ± sx	X ± sx	X ± sx		
**Chlortetracycline**	nd	nd	nd	≤200	waiting period 6 days
**Erythromycin**	nd	nd	nd	≤150	waiting period 4 days
**Neomycin**	na	na	na	≤500	for this type of antibiotic, no waiting period is required
**Oxytetracycline**	nd	nd	nd	≤200	waiting period 4 days
**Tetracycline**	nd	nd	nd	≤200	waiting period 4 days
**Tiamulin**	na	na	na	≤1000	for this type of antibiotic, no waiting period is required
**Tylosin**	nd	nd	nd	≤200	waiting period 4 days

### 4.3 The egg quality and safety characteristics

The egg quality and safety characteristics at units A, B, and C comply with the reference values established by the European Food Safety Authority (EFSA) (BIOHAZ, 2014) and Regulation 589/2008 [22]. This suggests that these units employ efficient management and monitoring systems.

The relative humidity (RH) in all three units examined falls within the optimal range of 70–80 %, in accordance with the recommendations specified in the EFSA study of 2014. Furthermore, this suggests that the eggs remain fresh and of high quality during the whole testing period.

The pH level of both the yolk and white plays a crucial role in determining the quality and freshness. The pH levels of the yolk exhibit variation, but they typically hover around the ideal range of 6 throughout all units. The pH of albumen displays significant variability, yet it consistently remains close to an optimal value. The observed discrepancy can be ascribed to natural fluctuations in the composition of eggs and the circumstances in which they are stored and handled prior to being examined.

Moreover, the temperature of the eggs is vital in maintaining their quality and freshness. According to Regulation 589/2008 [22], the temperatures recorded at all units fall within the range of 5–18 °C. Unit C displayed a higher average temperature, falling within the range of 5–18 °C. This suggests the need for more care to ensure proper storage conditions.

The obtained results underscore the importance of continuous monitoring and adherence to established standards to ensure the safety, quality, and freshness of eggs in-tended for human consumption (Table 9).The relative humidity in all three units fell within the optimal range (70–80 %), with the highest value recorded in unit C (79.1 %). The pH of yolk and white was consistent with reference values, showing minor variability.

**Table 9:** The egg quality and safety characteristics.

Parameter	Unit A	Unit B	Unit C	Reference values
	X ± sx	X ± sx	X ± sx	
**RH**	76.8 ± 1.33	73.2 ± 2.13	79.1 ± 1.02	70–80 % (BIOHAZ, 2014)
**pH yolk**	6.3 ± 2.14	6.2 ± 1.46	6.4 ± 1.62	6 (BIOHAZ, 2014)
**pH white**	7.8 ± 1.09	7.1 ± 2.03	7.2 ± 2.59	7.6 (BIOHAZ, 2014)
**Temparature**	5.6 ± 2.53	5.1 ± 1.36	7.2 ± 3.02	5–18 °C (Ord 589/2008)

Recent studies show that a significant segment of European consumers attach importance to animal welfare and the method of egg production, preferring eggs from cage-free hens [23]. This may influence product acceptance on the market and suggests the need for transparency in production practices.

### 4.4 The contamination of the eggs

The monitoring results from units A, B, and C (Table 10) confirm adherence to the predetermined threshold limits for various pollutants that could potentially be found in eggs meant for human consumption. This showcases the application of effective strategies for managing the presence of substances that could be harmful to customers and the safety of food. Chemical contamination assessments (Table 10) revealed that dioxin levels were well below the permissible limit of 2.5 pg/g fat, with the lowest value observed in unit B (1.2 pg/g fat). Similarly, non-dioxin-like PCB levels were within acceptable limits across all units.

**Table 10:** Contamination from the 3 egg sorting and packaging stations.

Parameter	Unit A	Unit B	Unit C	Reference values
	X ± sx	X ± sx	X ± sx	
Sum of dioxins (pg WHO- PCDD/F- TEQ/g)	1.7 ±0 .03	1.2 ± 0.21	1.6 ± 0.02	2,5 pg/g fat (2023/Ord 915)
Sum of dioxins and dioxin-like PCBs (pg WHO-PCDD/F-PCB-TEQ/g)	2.6 ± 0.34	3.2 ± 1.29	3.4 ± 1.25	5,0 pg/g fat (2023/Ord 915)
Sum of non dioxin-like PCBs (ng/g)	28 ± 1.32	27.1 ± 2.38	30.2 ± 2.61	40 ng/g fat [31]
Sum of PFOS, PFOA, PFNA and PFHxS (Perfluoroalkyl and polyfluoroalkyl substances)	0.9 ± 0.34	0.2 ± 0.21	0.3 ± 0.07	1.7(2023/Ord 915)
Melamine	1.9 ± 0.05	2.1 ± 1.36	1.9 ± 1.01	2.5 mg/kg (2023/Ord 915)
Fipronil (sum Fipronil + Fipronil sulfone)	< LOD	<LOD	<LOD	LOD = 0.005 mg/kg (2005/Ord 396)

X - the average of the determined value; sx - the standard deviation; LOD – the limit of detection.

The cumulative amounts of dioxins and dioxin-like PCBs detected in eggs obtained from all units are determined to be lower than the reference criterion of 5.0 pg/g fat. Unit A has the minimal average concentration compared to all the other units. The quantities of non-dioxin-like PCBs detected in all units were well below the reference limit of 40 ng/g fat. This suggests that the management procedures are successfully executed, hence preventing contamination with these persistent organic pollutants. The results of the study indicate that the levels of dioxins and PCBs in the eggs analyzed are below the maximum limits allowed by European regulations. This is essential given the ongoing concerns about chemical contaminants in food products.

The levels of perfluoroalkyl and poly-fluoroalkyl chemicals (PFOS, PFOA, PFNA, and PFHxS) found in eggs from the examined units are far lower than the reference value of 1.7, suggesting that there is very little risk to human health. These pollutants are a concern because they persist for a long time and have the potential to negatively impact human health. Unit B demonstrates the lowest average concentration, suggesting the successful implementation of effective management practices.

Previous research has highlighted the contamination of eggs with *Salmonella* in various production systems [23]. The present study, by carefully monitoring temperature and humidity during storage and transport, contributes to reducing the risk of contamination, aligning with the conclusions of other research that emphasizes the importance of controlling environmental conditions.

To meet consumer expectations and ensure competitiveness in the international market, producers should adopt production standards that promote animal welfare and food safety [24].

### 4.5 Comparison of current procedures and proposed PRP and HACCP plans

The current procedures employed in the analyzed units adhere to GPFH, FSSC 22000 V6, and IFS Food V8 standards, ensuring compliance with existing food safety regulations. However, the proposed PRP and HACCP plans introduce additional layers of preventive and corrective measures that aim to address both procedural gaps and enhance the overall robustness of the safety protocols.

### 4.6 Risk identification and management

Current methods primarily focus on compliance with predefined thresholds (e.g., temperature, humidity, and contamination limits) without a structured approach to proactive risk identification. In contrast, the proposed plans incorporate detailed hazard assessments using the HACCP framework, which identifies critical control points (e.g., storage and transport conditions) and implements specific corrective actions (e.g., rapid cooling for eggs stored beyond 18 °C for extended periods).

### 4.7 Integration of preventive measures

Proposed PRP plans emphasize preventive measures, such as.

- Continuous monitoring of environmental parameters using advanced technologies (e.g., IoT sensors).
- Enhanced hygiene protocols to address discrepancies observed in unit C, including more frequent sanitation schedules and stricter staff training programs.

### 4.8 Traceability and transparency

While current practices ensure basic traceability, the proposed plans include advanced tracking systems and detailed documentation at every stage of production, enabling faster responses to potential safety incidents and fostering consumer trust.

### 4.9 Demonstrated advantages

Simulation data and pilot studies indicate that the proposed plans reduce the risk of microbial growth by an additional 15 %, improve the consistency of egg quality parameters across units, and enhance the overall reliability of the food safety system.

## 5 Conclusions

This study evaluated the risks and hazards associated with egg sorting and packaging processes, demonstrating compliance with GPFH, FSSC 22000 V6, and IFS Food V8 standards. *Salmonella* spp. was identified as the main significant risk, and the implementation of advanced preventive measures, including strict monitoring of temperature (5–18 °C) and humidity (70–80 %), confirmed their effectiveness in controlling microbial contamination and maintaining egg quality. While minor discrepancies in cleanliness were observed in unit C, overall compliance with EU standards was achieved. The FSSC and IFS systems provide robust tools for monitoring food fraud, food defense, and product traceability, supporting both processing units and researchers through technology transfer. Recommendations include improving hygiene standards, intensifying staff training programs, and increasing transparency for consumers regarding traceability and production practices. Future research should focus on analyzing the impact of storage parameters on egg freshness and studying consumer preferences for eggs from different rearing systems, contributing to process optimization and enhancing the industry's competitiveness.

## CRediT authorship contribution statement

**Ioana Cristina Crivei:** Writing – original draft, Project administration, Methodology, Investigation, Formal analysis, Data curation, Conceptualization. **Romina Alina Marc:** Writing – review & editing, Writing – original draft, Visualization, Validation, Supervision, Formal analysis, Conceptualization. **Roxana Nicoleta Rațu:** Writing – original draft, Resources, Conceptualization. **Crina Carmen Mureșan:** Writing – review & editing, Visualization, Validation, Supervision. **Alina Narcisa Postolache:** Methodology, Investigation, Funding acquisition, Formal analysis, Conceptualization. **Florina Stoica:** Writing – original draft, Software, Formal analysis, Data curation. **Ionuț Dumitru Veleșcu:** Writing – original draft, Methodology, Investigation, Formal analysis. **Aida Albu:** Writing – original draft, Formal analysis, Data curation.

## Data and code availability statement

No new data was generated for the research described in the article.

## Funding

This research did not receive any specific grant from funding agencies in the public, commercial, or not-for-profit sectors.

## Declaration of competing interest

The authors declare the following financial interests/personal relationships which may be considered as potential competing interests: Ioana Cristina Crivei reports financial support was provided by "Ion Ionescu de la Brad" University of Life Sciences, Iași. Ioana Cristina Crivei reports a relationship with "Ion Ionescu de la Brad" University of Life Sciences, Iași that includes: board membership. If there are other authors, they declare that they have no known competing financial interests or personal relationships that could have appeared to influence the work reported in this paper.
